# Risk analysis for subsequent fracture of osteoporotic fractures in Chinese women over age 60: a nationwide cross-sectional study

**DOI:** 10.1038/s41598-024-64170-w

**Published:** 2024-06-10

**Authors:** Nan Tang, Ling Gao, Jie Song, Yeyuan Li, Mi Song, Chen Qiu, Mengqi Shao, Jingru Chen, Shan Li, Qingmei Wang, Qingqing Su, Yuan Gao

**Affiliations:** 1 PLA Medical School, PLA General Hospital, Beijing, 100853 China; 2 Department of Nursing, 1th Medical Center, PLA General Hospital, Beijing, 100853 China; 3 Beijing Haidian District Wanshou Road Community Health Service Center, Beijing, 100017 China; 4 Central Patient Management Department, 1th Medical Center, PLA General Hospital, No. 28 Fuxing Road, Beijing, 100853 China

**Keywords:** Osteoporosis, Osteoporotic fractures, Subsequent fractures, Older women, Fracture repair, Risk factors

## Abstract

Prevention of subsequent fracture is a major public health challenge in the field of osteoporosis prevention and treatment, and older women are at high risk for osteoporotic fractures. This study aimed to examine factors associated with subsequent fracture in older Chinese women with osteoporosis. We collected data on 9212 older female patients with osteoporotic fractures from 580 medical institutions in 31 provinces of China. Higher odds of subsequent fractures were associated with age of 70–79 years (OR 1.218, 95% CI 1.049–1.414), age ≥ 80 (OR 1.455, 95% CI 1.222–1.732), index fracture site was vertebrae (OR 1.472, 95% CI 1.194–1.815) and hip (OR 1.286, 95% CI 1.041–1.590), index fracture caused by fall (OR 1.822, 95% CI 1.281–2.591), strain (OR 1.587, 95% CI 1.178–2.139), no inducement (OR 1.541, 95% CI 1.043–2.277), and assessed as high risk of fracture (OR 1.865, 95% CI 1.439–2.416), BMD T-score ≤ −2.5 (OR 1.725, 95% CI 1.440–2.067), history of surgery (OR 3.941, 95% CI 3.475–4.471) and trauma (OR 8.075, 95% CI 6.941–9.395). Low risk of fall (OR 0.681, 95% CI 0.513–0.904), use of anti-osteoporosis medication (AOM, OR 0.801, 95% CI 0.693–0.926), and women who had received fall prevention health education (OR 0.583, 95% CI 0.465–0.730) associated with lower risk. The areas under the curve of the prediction model was 0.818. The sensitivity was 67.0% and the specificity was 82.0%. The prediction model showed a good ability to predict the risk of subsequent fracture in older women with osteoporotic fractures and are suitable for early self-measurement which may benefit post-fracture management.

## Introduction

Osteoporotic fractures (OPFs), also known as fragility fractures, are fractures that occur after low-energy external forces due to increased bone fragility caused by decreased bone mass and destruction of the bone microstructure^1^. WHO data show that, globally, one-third of women and one-fifth of men over the age of 50 suffer one or more osteoporotic fractures, resulting in severe disability and 20% death rate within one year, and a 50% permanent disability rate^2^. Women are more likely than men to develop osteoporosis 5–10 years after menopause due to estrogen deficiency^3^. The incidence of hip fracture is twice as high in older women as in men^4^. The prevalence of osteoporosis is highest among postmenopausal women in the older population, with a prevalence of 51.6% in women over 65 years of age^5^. In recent years, some progress has been made in the orthopaedic treatment of osteoporotic fractures, but the prevention of subsequent fracture is still unsatisfactory. The main reason is that only 6.5% of patients in China received medications for treatment of osteoporosis within 6 months after a fracture, and the majority of patients are exposed to an extremely high risk of subsequent fracture^6^.

In 2013, the International Osteoporosis Foundation (IOF) stated in its "Global Initiative to Break the Cycle of Osteoporotic Fractures" that 50% of patients with osteoporotic fractures will suffer a subsequent fracture^6^. The risk of subsequent fracture increases exponentially in patients with osteoporotic fractures^7,8^. Compared with the index fracture, subsequent fracture are more harmful, and increases the risk of death by 55%^9^. Numerous studies have confirmed that subsequent fracture after a major osteoporotic fracture occurs within 2 years of the fracture and that 2 years is the "imminent" risk period for subsequent fracture^10^. The risk of subsequent fracture is 2.7 times higher within the first year of the first fracture than in patients with no history of fracture, with 67.1% of subsequent fractures occurring in the first year, of which 51.1% occurred within 6 months of the initial fracture^11,12^. Therefore, it is important to develop predictive models to determine the risk of subsequent fracture and identify controllable factors as early as possible after a fracture.

The risk factors for osteoporosis have been widely studied^13^. The risk factors for subsequent fracture are complex and multidimensional^12^. A Swedish research included 242,108 patients with osteoporotic fractures showed that advanced age and spinal fracture were independent risk factors for subsequent fracture, and the risk of subsequent fracture in patients with spinal fracture was 2.72 times higher than in patients with hip fracture^14^. Population-based registries in Denmark have identified female sex, older age, excessive alcohol consumption, living alone, and fracture history as risk factors for a second hip fracture^15^. Some studies have also found interactions between individual common independent risk factors, for example, Awal et al. found a cumulative effect of gender, age and skeletal pathology on the occurrence of subsequent fracture^16^.

Risk factors for subsequent fracture of osteoporotic fractures vary among populations in different regions, and effective risk screening and prevention can reduce the incidence of subsequent fracture. The predictive models FRAX (Fracture risk assessment tool), QFracture, and Garvan, which have been developed based on population data from the USA, UK, and Australia respectively, are utilized for identifying the risk of osteoporotic fractures^17^. FRAX does not incorporate important factors such as falls and medications, which may underestimate fracture risk. QFracture contains 31 risk factors and is suitable for patients with multiple chronic diseases^17^. The applicability of these two models in the Chinese population needs to be further verified. Garvan is suitable for individuals at high risk of falls, but Garvan is not a good predictor of risk in postmenopausal women^18^. In addition, the OSTA (osteoporosis self-assessment tool for Asians) is a simple assessment tool for self-screening for osteoporosis in postmenopausal women. The tool uses age and weight to calculate a risk index that guides the patient in determining the need for BMD testing. However, this tool has few indicators and low specificity^19^. Another method is to use diagnostic techniques such as dual-energy X-ray bone densitometry and quantitative CT to predict the risk of osteoporosis and determine the risk of fracture and fracture-prone areas accordingly. The implementation of this method is constrained by the uneven distribution of medical resources and the limited emphasis on preventive measures.

Although these tools can predict fracture risk, their predictive efficacy in the Chinese population remains to be explored. In addition, there are insufficient studies on the risk factors of subsequent fracture, and few literatures can systematically investigate and analyze the influence of multiple factors on the occurrence of subsequent fracture in older adults. We focused on the key population of osteoporotic fractures in older women and explored the influencing factors of the occurrence of subsequent fracture. At the same time, based on Chinese patient data, a localized older female subsequent fracture influencing factor model was constructed to provide a basis for clinical implementation of accurate risk prediction, prevention and management of subsequent fracture.

## Results

### Characteristics of patients and disease

**Figure 1:**
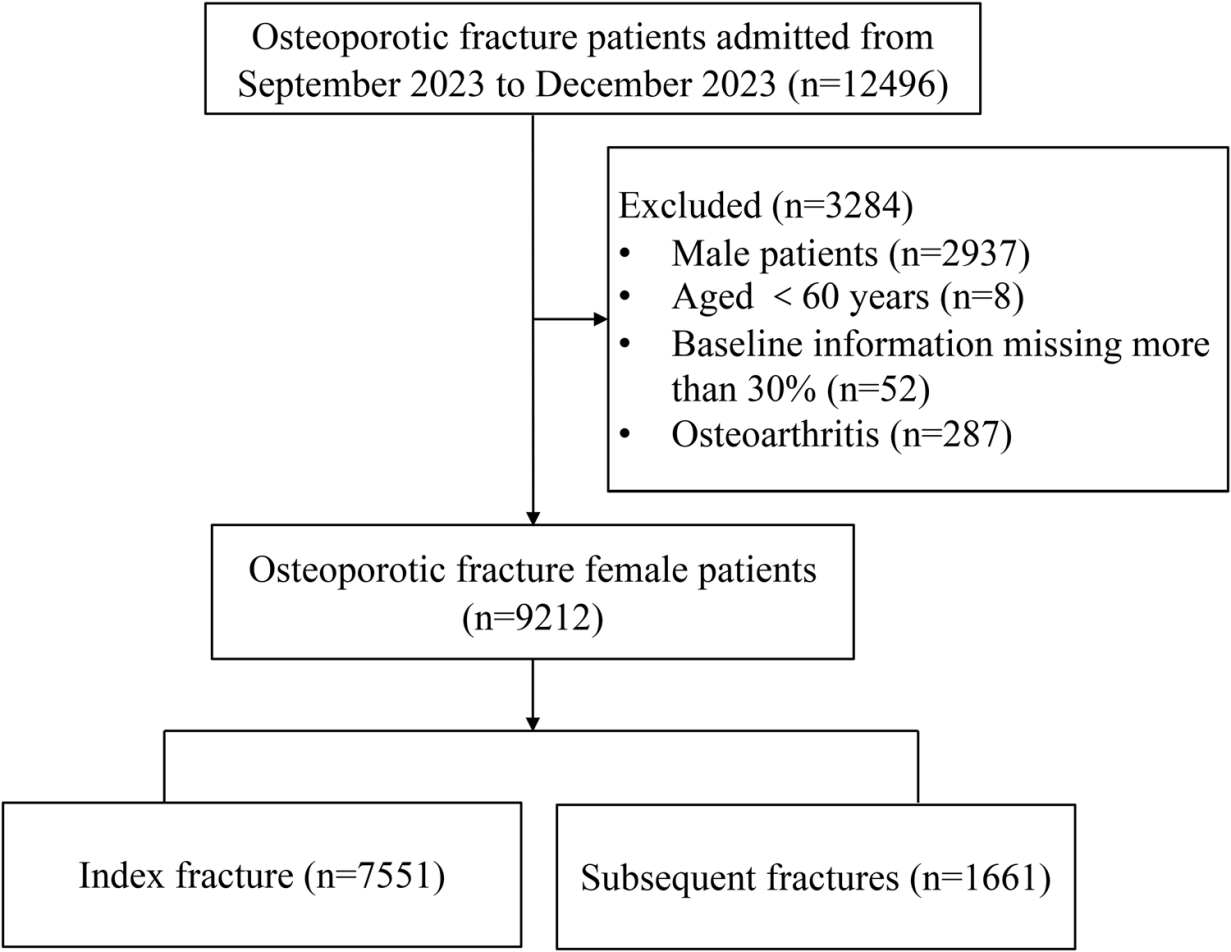
Flow chart.

A total of 9212 older female were included in this study (Fig. 1). Data were obtained from 594 medical institutions in 31 provinces of China, with 77.7% in tertiary hospitals (the highest grade according to the Hospital Classification and Management Standards) and the remaining 22.3% in secondary hospitals. Descriptive characteristics for the participants are summarized in Table 1. The mean age of the patients was (74 ± 8.449) years, of which 75.7% were under 80 years of age and 24.3% were above 80 years of age. The BMI of older women was 22.91 ± 3.63.

**Table 1:** General characteristics of the study population according to fracture.

Characters		Total n = 9212	Index Fracture n = 7551	Subsequent fractures n = 1661	*P*-value
General information					
Age (years; mean ± SD)		74 ± 8.449	73.65 ± 8.442	75.56 ± 8.306	< 0.001
BMI (mean ± SD)		22.91 ± 3.63	22.94 ± 3.58	22.78 ± 3.83	0.116
Comorbidity	Yes	6899(74.9%)	5575 (73.8%)	1324 (79.7%)	
	No	2313(25.1%)	1976 (26.2%)	337 (20.3%)	< 0.001
Surgical history	Yes	2957 (32.1%)	1873 (24.8%)	1084 (65.3%)	
	No	6255 (67.9%)	5678 (75.2%)	577 (34.7%)	< 0.001
Trauma history	Yes	1129 (12.3%)	447 (5.9%)	682 (41.1%)	
	No	8083 (87.7%)	7104 (94.1%)	979 (58.9%)	< 0.001
Family history	Yes	274 (3.0%)	201 (2.7%)	73 (4.4%)	
	No	8938 (97.0%)	7350 (97.3%)	1588 (95.6%)	< 0.001
Smoking history	Yes	115 (1.2%)	94 (1.2%)	21 (1.3%)	
	Quit smoking	51 (0.6%)	40 (0.5%)	11 (0.7%)	
	Never	9046 (98.2%)	7417 (98.3%)	1629 (98.0%)	0.803
Drinking history	Yes	82 (0.9%)	61 (0.8%)	21 (1.3%)	
	Quit drinking	40 (0.4%)	27 (0.4%)	13 (0.8%)	
	Never	9090 (98.7%)	7463 (98.8%)	1627 (98.0%)	0.011
Occupation	Unemployed	4196 (45.5%)	3868 (51.2%)	747 (45.0%)	
	Employed	5016 (54.5%)	3683 (48.8%)	914 (55.0%)	< 0.001
Education	Secondary school or below	7769 (84.3%)	6411 (84.9%)	1358 (81.8%)	
	High school	1028 (11.2%)	820 (10.9%)	208 (12.5%)	
	College and above	415 (4.5%)	320 (4.2%)	95 (5.7%)	0.003
Marital status	Married or partnered	7816 (84.8%)	6487 (85.9%)	1329 (80.0%)	
	Never married	31 (0.3%)	25 (0.3%)	6 (0.4%)	
	Divorced or widowed	1365 (14.9%)	1039 (13.8%)	326 (19.6%)	< 0.001
Number of children	0	60 (0.7%)	50 (0.6%)	10 (0.6%)	
	1	1771 (19.2%)	1495 (19.8%)	276 (16.6%)	
	2	3618 (39.3%)	3017 (40.0%)	601 (36.2%)	
	≥ 3	3763 (40.8%)	2989 (39.6%)	774 (46.6%)	< 0.001
Family income (thousand yuan/year)	< 10	5985 (65.0%)	4892 (64.8%)	1093 (65.8%)	
	10–20	2347 (25.5%)	1956 (25.9%)	391 (23.6%)	
	20–30	556 (6.0%)	446 (5.9%)	110 (6.6%)	
	> 30	324 (3.5%)	257 (3.4%)	67 (4.0%)	0.117
Characteristics of index fracture					
Fracture site	Vertebral	4186 (45.5%)	3299 (43.7%)	887 (53.4%)	
	Hip	3393 (36.8%)	2803 (37.1%)	590 (35.5%)	
	Other	1633 (17.7%)	1449 (19.2%)	184 (11.1%)	< 0.001
Cause of fracture	Fall	6346 (68.9%)	5263 (69.7%)	1083 (65.2%)	
	Slight strike	780 (8.5%)	691 (9.2%)	89 (5.4%)	
	Strain	1332 (14.4%)	1050 (13.9%)	282 (17.0%)	
	No inducement	329 (3.6%)	245 (3.2%)	84 (5.1%)	
	Other	425 (4.6%)	302 (4.0%)	123 (7.4%)	< 0.001
Prevention information after index fracture					
BMD T-score	≤ −2.5	3362 (36.5%)	2539 (33.6%)	823 (49.5%)	
	> −2.5	1957 (21.2%)	1702 (22.5%)	255 (15.4%)	
	Unknown	3893 (42.3%)	3310 (43.8%)	583 (35.1%)	< 0.001
Fall risk assessment	Not evaluated	5764 (62.6%)	4814 (63.8%)	950 (57.2%)	
	Low risk	641 (7.0%)	562 (7.4%)	79 (4.8%)	
	Moderate risk	759 (8.2%)	604 (8.0%)	155 (9.3%)	
	High risk	2048 (22.2%)	1571 (20.8%)	1469 (88.4%)	< 0.001
Fracture risk assessment	Not evaluated	8613 (93.5%)	7138 (94.5%)	1475 (88.8%)	
	Low risk	39 (0.4%)	34 (0.5%)	5 (0.3%)	
	Moderate risk	93 (1.0%)	77 (1.0%)	16 (1.0%)	
	High risk	467 (5.1%)	302 (4.0%)	165 (9.9%)	< 0.001
Walking aids	No	3995 (43.4%)	3337 (44.2%)	658 (39.6%)	
	Yes	5217 (56.6%)	4214 (55.8%)	1003 (60.4%)	0.001
Use of AOM	No	4822 (52.3%)	3835 (50.8%)	987 (59.4%)	
	Yes	4390 (47.7%)	3716 (49.2%)	674 (40.6%)	< 0.001
Use of calcium or vitamin D	No	6555 (71.2%)	5266 (69.7%)	1289 (77.6%)	
	Yes	2657 (28.8%)	2285 (30.3%)	372 (22.4%)	< 0.001
Fall prevention health education	No	724 (7.9%)	572 (7.6%)	152 (9.2%)	
	Yes	8488 (92.1%)	6979 (92.4%)	1509 (90.8%)	0.031

*BMI* body mass index, *BMD* bone mineral density, *AOM* anti-osteoporosis medication.

There were 7551 patients with index fractures and 1661 with subsequent fractures. Compared with index fracture, subsequent fracture patients were on average older and had a higher number of comorbidity (*P* < 0.001). Patients in the subsequent fracture group had a higher percentage of surgical history, trauma history and family history than those in the first fracture group (*P* < 0.001). There was no statistically significant difference between the two groups in the comparison of smoking history (*P* = 0.803), while the percentage of alcohol consumption in the subsequent fracture group was more than that in the index fracture group (*P* = 0.011). In addition, the differences between two groups were significant in terms of occupation, educational, marital status, and number of children. There was no statistically significant difference between groups in terms of family income (*P* = 0.117).

Severe osteoporosis was diagnosed by BMD in 36.5% of patients. The highest percentage of fracture site was vertebral (45.5%), followed by hip (36.8%). Due to fall, 68.9% of the patients had fracture. Anti-osteoporosis medication (AOM) was applied to 47.7% of the patients, and complications occurred in 4.4% of the patients. The differences between the two groups were significant when comparing fracture site, cause of fracture, severity of osteoporosis, use of AOM, complication rate, and self-care ability (*P* < 0.001).

With regard to the prevention of subsequent fractures, 37.4% of patients were assessed for risk of falls and only 6.7% for risk of fracture. After a fracture 56.6% of patients used crutches, walkers or wheelchairs, 28.8% of women used calcium or vitamin D. There were significant differences between the two groups in the use of mobility aids, calcium or vitamin D, medication adherence, and receipt of fall prevention education (Table 1).

### Risk factors of subsequent fracture

The univariate analyses of the association between independent variables and subsequent fracture in Table 2. The results found that the factors, including age, BMI, comorbidity, surgical history, trauma history, drinking history, occupation, education, marital status, number of children, and self-care ability were related to the risk of subsequent fracture.

**Table 2:** Results of monofactor logistic regression analysis of risk factors for subsequent fractures.

Variable		OR (95% CI)	*P*-value
Age (years; mean ± SD)		1.027 (1.020–1.033)	< 0.001
Comorbidity	No	Reference	
	Yes	1.393 (1.223–1.586)	< 0.001
Surgical history	No	Reference	
	Yes	5.569 (5.083–6.381)	< 0.001
Trauma history	No	Reference	
	Yes	11.071 (9.657–12.693)	< 0.001
Drinking history	Never	Reference	
	Quit drinking	2.209 (1.137–4.289)	0.019
	Yes	1.579 (0.959–2.600)	0.073
Occupation	Unemployed	Reference	
	Employed	0.778 (0.699–0.866)	< 0.001
Education	Secondary school or below	Reference	
	High school	1.197 (1.017–1.410)	0.030
	College and above	1.402 (1.106–1.775)	0.005
Marital status	Married or partnered	Reference	
	Never married	1.171 (0.480–2.861)	0.728
	Divorced or widowed	1.532 (1.334–1.758)	< 0.001
Number of children	≥ 3	Reference	
	2	0.769 (0.684–0.866)	< 0.001
	1	0.713 (0.613–0.829)	< 0.001
	0	0.772 (0.390–1.530)	0.459
Fracture site (prior)	Other	Reference	
	Vertebral	2.117 (1.786–2.511)	< 0.001
	Hip	1.658 (1.388–1.979)	< 0.001
Cause of fracture (prior)	Slight strike	Reference	
	Fall	3.162 (2.332–4.287)	< 0.001
	Strain	2.085 (1.613–2.696)	< 0.001
	No inducement	2.662 (1.910–3.710)	< 0.001
	Other	1.598 (1.269–2.011)	< 0.001
BMD T-score	> -2.5	Reference	
	≤ -2.5	2.163 (1.856–2.522)	< 0.001
	Unknown	1.176 (1.003–1.377)	0.045
Fall risk assessment	Not evaluated	Reference	
	Low risk	0.712 (0.557–0.911)	0.007
	Moderate risk	1.300 (1.076–1.572)	0.007
	High risk	1.539 (1.359–1.741)	< 0.001
Fracture risk assessment	Not evaluated	Reference	
	Low risk	0.712 (0.278–1.823)	0.478
	Moderate risk	1.006 (0.585–1.728)	0.984
	High risk	2.644 (2.169–3.222)	< 0.001
Walking aids	No	Reference	
	Yes	1.207 (1.083–1.345)	0.001
Use of AOM	No	Reference	
	Yes	0.705 (0.633–0.785)	< 0.001
Use of calcium or vitamin D	No	Reference	
	Yes	0.665 (0.587–0.754)	< 0.001
Fall prevention health education	No	Reference	
	Yes	0.814 (0.675–0.981)	0.031

Table 3 shows the results of multivariate logistic regression analyses of subsequent fracture based on univariate analyses results. Multivariate analysis showed that age, index fracture site, index fracture causes, risk assessment, BMD T-score ≤ −2.5, AOM use, fall prevention health education, surgical history, trauma history were associated with subsequent fracture. Individuals aged 70–79 years (OR 1.218, 95% CI 1.049–1.414) or aged ≥ 80 (OR 1.455, 95% CI 1.222–1.732) had a higher likelihood of experiencing subsequent fracture. Index fracture site of vertebrae (OR 1.472, 95% CI 1.194 to 1.815) and hip (OR 1.286, 95% CI 1.041–1.590) lead to higher risk of subsequent fracture. Index fracture of women caused by fall (OR 1.822, 95% CI 1.281–2.591), strain (OR 1.587, 95% CI 1.178–2.139), no inducement (OR 1.541, 95% CI 1.043–2.277) were more likely to suffer from subsequent fractures. Higher odds of subsequent fractures were also associated with high risk of fracture (OR 1.865, 95% CI 1.439–2.416), BMD T-score ≤ −2.5 (OR 1.725, 95% CI 1.440–2.067), history of surgery (OR 3.941, 95% CI 3.475–4.471) and trauma (OR–8.075, 95% CI 6.941–9.395). Patients who assessed as low risk of fall (OR 0.681, 95% CI 0.513–0.904), use of AOM (OR 0.801, 95% CI 0.693–0.926), and had received fall prevention health education (OR 0.583, 95% CI 0.465–0.730) were less likely to suffer from subsequent fractures.

**Table 3:** Results of multivariate logistic regression analysis of risk factors for subsequent fractures.

Variable		OR (95% CI)	*P*-value
Age	< 70	Reference	
	70–79	1.218 (1.049–1.414)	0.010
	≥ 80	1.455 (1.222–1.732)	< 0.001
Surgical history	No	Reference	
	Yes	3.941 (3.475–4.471)	< 0.001
Trauma history	No	Reference	
	Yes	8.075 (6.941–9.395)	< 0.001
Fracture site (prior)	Other	Reference	
	Vertebral	1.472 (1.194–1.815)	< 0.001
	Hip	1.286 (1.041–1.590)	0.020
Cause of fracture (prior)	Slight strike	Reference	
	Fall	1.822 (1.281–2.591)	0.001
	Strain	1.587 (1.178–2.139)	0.002
	No inducement	1.541 (1.043–2.277)	0.030
	Other	1.203 (0.925–1.565)	0.168
BMD T-score	> -2.5	Reference	
	≤ -2.5	1.725 (1.440–2.067)	< 0.001
	Unknown	1.039 (0.868–1.245)	0.676
Fall risk assessment	Not evaluated	Reference	
	Low risk	0.681 (0.513–0.904)	0.008
	Moderate risk	1.163 (0.923–1.564)	0.201
	High risk	0.959 (0.817–1.125)	0.607
Fracture risk assessment	Not evaluated	Reference	
	Low risk	1.633 (0.592–4.505)	0.344
	Moderate risk	1.206 (0.635–2.290)	0.567
	High risk	1.865 (1.439–2.416)	< 0.001
Use of AOM	No	Reference	
	Yes	0.801 (0.693–0.926)	0.003
Fall prevention health education	No	Reference	
	Yes	0.583 (0.465–0.730)	< 0.001

### Construction of subsequent fracture risk prediction model and effect analysis

**Figure 2:**
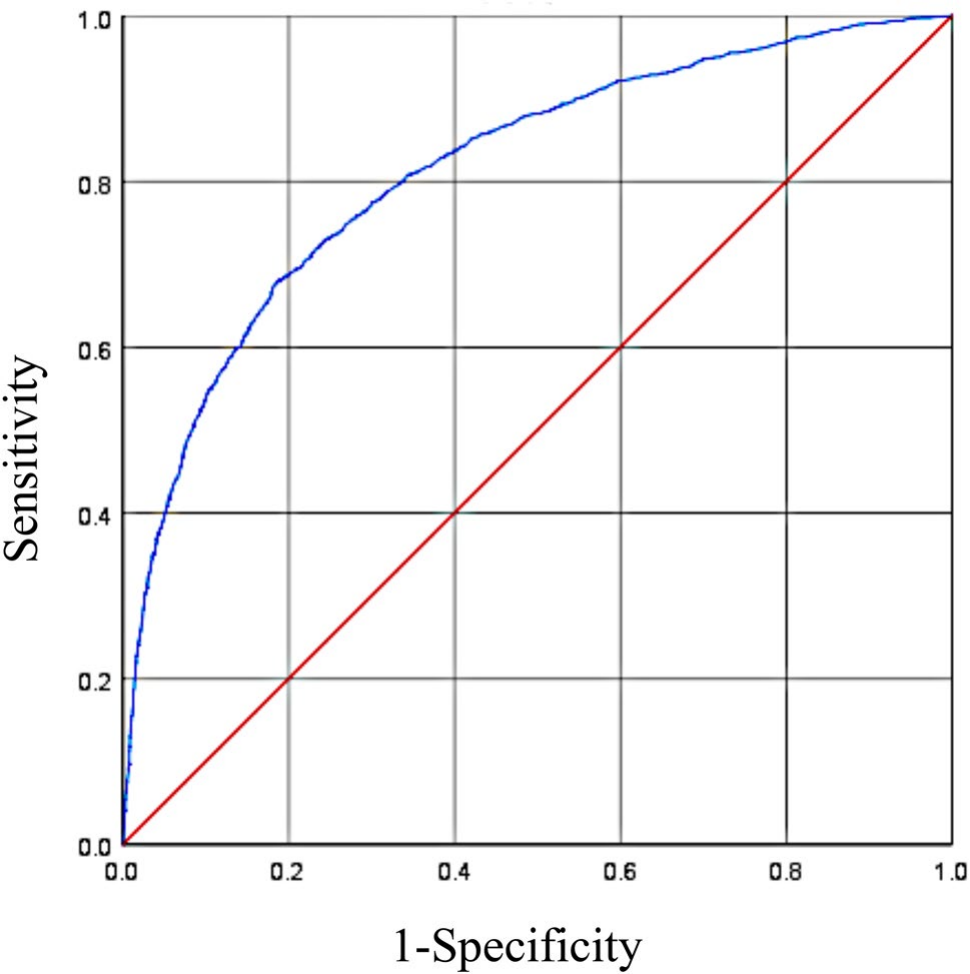
ROC curve for risk of subsequent fracture.

The risk prediction model was constructed using the results of multivariate analysis as predictors. The model demonstrated good predictive power with an area under the ROC curve (AUC) of 0.818 (95% CI 0.806–0.829), *p* < 0.001 (Fig. 2). The maximum value of Youden index was used to determine the optimal cutoff value of the prediction model. The maximum Youden index of the ROC curve was 0.494, and the optimal cutoff value was 0.207, in which the sensitivity was 67.0% and the specificity was 82.0%.

## Discussion

Preventing subsequent fracture is a priority in reducing the harms of osteoporosis. 20% of patients with osteoporotic fracture will have a subsequent fracture within 1 year, which is 55% higher than the risk of death from the initial fracture, and 6.2 times higher than the economic burden^20,21^. The occurrence of subsequent fracture is detrimental to fracture healing, rehabilitation, self-care ability, and psychological expectations. At the same time, subsequent fracture leads to an increase in disability, incapacity, mortality and a decrease in the quality of life of the older population^22^. Taking hip fracture as an example, the morbidity and mortality rate within 1 year after fracture is as high as 36%, of which more than 70% of patients die due to subsequent fracture^2^. In our study, age, index fracture site, index fracture cause, fracture risk assessment and fall risk assessment, BMD T-score, AOM use, fall prevention health education, surgical history, trauma history were found to be independent risk factors for the occurrence of subsequent fracture.

The incidence of subsequent fractures in older women in our study was 18%. This result is lower than the findings of Yang et al. in OP patients over 65 years of age in Hebei, China (3-year postoperative subsequent fracture rate of 25.96%)^23^. However, the incidence of subsequent fracture in all-age OP in a meta-analysis was 13.4%^24^. Compared with all-age OP, the incidence of subsequent fracture in older females of our study is clearly higher. Consistent with our study, in a French cohort study of 560,499 patients with OF analyzed retrospectively, the incidence of subsequent fracture in women who experienced at least one subsequent fracture that resulted in hospitalisation was 17.9%^11^. The American Association of Clinical Endocrinologists (AACE) and American College of Endocrinology (ACE) 2020 edition of the Clinical Practice Guidelines for the Management of Postmenopausal Osteoporosis specify the definition of patients at very high risk of fracture and pharmacologic treatment options. For the prevention of subsequent fracture in postmenopausal women, the guidelines recommend standardized OP treatment, full follow-up, and monitoring of efficacy and adherence^25^. However, risk assessment methods for subsequent fracture have not been mentioned, and more research is urgently needed to optimize the risk assessment and management of subsequent fracture in this priority population.

It has been suggested that age is an important factor in increasing the risk of morbidity for all types of osteoporotic fractures^13^. The results of a retrospective study suggest that the effects of oestrogen deficiency on bone loss and impaired microstructure of bone tissue in postmenopausal women are more severe with age and that the risk of subsequent fracture increases with age^3^. Our study also validates this idea. senior older women over 80 years of age bear a greater risk of subsequent fracture than patients in the < 70 and 70–79 age groups. The magnitude of the risk of subsequent fracture varies depending on the location of the previous fracture and varies. Our study found a higher risk of subsequent fracture in vertebral fractures than in patients with hip fractures and fractures at other sites. Several cohort studies have found that patients with vertebral fractures have the highest risk of subsequent fracture among the types of previous fracture, and the lowest risk after tibia/fibula and ankle fractures^2,26^. Lindsay et al.^27^ suggested that patients with vertebral fractures have a five times higher incidence of secondary vertebral fractures than patients with no history of previous fracture. Our result suggests that the site of fracture is one of the important factors in the assessment of the risk of subsequent fracture. Patients with vertebral fractures should also be included in the focus of attention in the prevention of subsequent fracture.

Second, in our results, patients with fractures caused by falls, strain, no inducement had a greater risk of subsequent fractures than those caused by slight strike. Among these causes, falls accounted for the largest proportion of subsequent fractures (65.2%), and falls were controllable factors. Several guidelines state that falls are an independent risk factor for subsequent fracture^28^; and age and medication-induced imbalances also increase the risk of fracture from falls^7^. We found that patients who underwent assessment of low fall risk were less likely to suffer from subsequent fractures, suggesting that timely assessment of fall risk after fracture can be used as a means of subsequent fracture prevention. The 2013 UK Fracture Liaison Services (FLS) Code of Practice for the management of falls suggests that falls risk assessment is central to the prevention and treatment of subsequent fractures. Assessing falls risk and preventing falls is now available as a non-pharmacological intervention programme for the prevention of subsequent fractures^8^. Also, our study confirms that falls prevention health education is a protective factor in the occurrence of subsequent fracture. Multinational studies and consensus suggest that important non-pharmacological interventions for OF include fall risk assessment and health education to help patients understand the severity of subsequent fracture, the optimal time to prevent it, preventive and curative measures, and functional exercise^29^.

BMD is a non-invasive test that reflects bone mineral content, and a T-score ≤ −2.5 is the basis for osteoporosis diagnosis. The guideline indicates that T-score ≤ −2.5 with fracture is considered severe osteoporosis, and in China, BMD testing has been included in the routine physical examination of people over 40 years of age^30^. We found that patients with T-score ≤ −2.5, assessed as high risk of fracture, and those not using AOM had a higher risk of subsequent fracture. In our study 49.2% of OPF patients and 40.6% of subsequent fractures patients were treated with AOM, 93.3% were not assessed for fracture risk. This is a gap with the recommended strategies for the care of women with osteoporotic fractures in the guidelines^5^. Guidelines recommend that women over 65 years of age with pre-existing fragility fractures be considered for treatment without further evaluation^5^. In younger postmenopausal women, BMD test results can be used as a standard for medication^5^. Multiple consensus recommends that patients treated with anti-osteoporotic medications after a fracture should undergo annual BMD testing to assess fracture risk^28,31,32^. At present, unclear management responsibilities of subsequent fracture prevention and treatment lead to low risk assessment and treatment rates^33^. Secondly, many orthopaedic doctors still have a weak concept of prevention and management of subsequent fracture after osteoporotic fracture^33^. Moreover, patients’ concerns about the cost of diagnosis and treatment, adverse drug reactions and efficacy led to poor compliance with the management of subsequent fracture prevention and treatment^34^. Therefore, more effective strategies are urgently needed to regulate post-fracture management and reverse the disease burden of osteoporosis.

Patients with a history of trauma and surgery are at greater risk of subsequent fracture. The risk of subsequent fracture was 8.15 times higher in patients with a history of trauma than in those without a history of trauma. Siris et al.^35^ found that postmenopausal women with a history of previous fracture were more prone to secondary fracture and the risk increased with the number of previous fractures. Previous trauma leads to disruption of the bone structure and changes in the structure of the bone cortex and trabeculae, which in turn leads to a decrease in bone strength^36^. The risk of subsequent fracture in patients with a history of surgery is 3.98 times higher than in patients without a history of surgery. For example, percutaneous vertebroplasty (PVP) and percutaneous kyphoplasty (PKP) have a high incidence of secondary fractures after surgery^22^. Inadequate cement injection and excessive number of operated vertebrae lead to increased and unevenly distributed loading stresses on adjacent vertebrae, resulting in subsequent fracture^37^. Comorbidities and economic status were not included in the final model in this study, probably due to their correlation. Evidence suggests that patients with poor economic level have a tendency to have greater multimorbidity^38^. And economic level was correlated with patient frailty. Whereas high BMI and malnourishment are both associated with comorbidities and economic level^38^. Whether there is a mediating effect between these factors and fracture risk remains to be confirmed. In addition, the effect of alcohol consumption on subsequent fracture was not significant, probably due to the lower alcohol consumption of female patients in our study with a mean of 53.66 ± 18.890 ml/d. Guidelines indicate that excessive alcohol consumption is associated with increased fracture risk, where alcohol intake of more than 3 units/d (90 ml/d of spirits) is considered excessive^30^.

The principle of rehabilitation in the management of subsequent fracture is to balance two factors "promoting fracture healing" and "preventing bone loss". It is necessary to prevent premature rehabilitation training from affecting fracture healing, as well as complications such as bone loss, disuse induced muscle atrophy, pneumonia, and lower limb venous thrombosis caused by long-term immobilization. Therefore, OPF patients should be predicted for subsequent fracture risk as early as possible on the basis of standardized treatment, and high-risk groups should be screened for individualized rehabilitation interventions.

In comparison, the prediction model constructed in our study covers 10 pre-discharge static indicators, and the information is easy to obtain and simple to operate. It has been internally validated to show good discriminatory performance and is expected to form a clinically usable tool after further validation and optimisation. Although we included a large national sample, limitations remain. First, we did not collect complete surgical treatment modalities. Although some studies have shown a high incidence of secondary fractures after percutaneous vertebro plasty (PVP) and percutanouskyphoplasty (PKP) for osteoporotic vertebral compression fracture (OVCF)^22^. Secondly, self-reported form of data collection may be subject to recall bias, leading to possible inaccurate BMD reporting results and the time difference between subsequent and index fractures. Therefore, registration and long-term follow-up of patients with osteoporotic fractures is relevant to explore the pattern of subsequent fracture occurrence. Third, this data from 31 provinces of the country were not followed up, limiting our study to the analysis of out-of-hospital risk factors in this population. Fourth, this study was limited to inpatients and did not include patients attending outpatient clinics, which may have led to an underestimation of the incidence of subsequent fractures. To address these shortcomings, a subsequent fracture cohort could be established in the future to further explore the effects of surgical approach, type of trauma, BMD, nutrition, and health behaviours on subsequent fractures and time to occurrence. Furthermore, this study demonstrated that patients with AOM had a lower risk of subsequent fracture. This suggests that we should focus on the effects of AOM dose, duration of treatment and compliance on the occurrence of subsequent fractures. In the future, we will externally validate the model and develop user-friendly tools such as mobile phones or web application to enhance the promotional value of the model.

In this study, we collected data based on patient self-report, and 10 independent risk factors were included in the final prediction model, including age, index fracture site, index fracture cause, fracture risk assessment and fall risk assessment, BMD T-score, AOM use, fall prevention health education, surgical history, trauma history. These risk factors are easy to collect and help older adults self-assess and identify subsequent fracture risk. The results of this study highlight the critical importance of better managing older women with osteoporotic to improve screening for high risk of subsequent fracture as well as developing effective strategies to reduce the risk of subsequent fracture. This study provides clinicians and patients with an easy subsequent fracture risk assessment tool that is expected to yield benefits in reducing subsequent fracture incidence and osteoporotic mortality.

## Methods

### Study design and data collection

This national study was conducted by the Orthopedic Nursing Committee of Chinese Nursing Association. In order to take into account factors such as regional distribution and regional economy, 580 medical institutions in 31 provinces of China were selected by multistage sampling method for data collection. From September 2023 to December 2023, we included data on 9212 older women hospitalized for osteoporotic fractures.

The method of multistage sampling was adopted in our study. The first stage, we divided into four regions, northeast, east, central and west, according to the division of China's economic regions. In accordance with the principle of geographic decentralization and economic diversity, a number of cities were selected from each region and all provinces were covered. In the second stage, 2 to 3 hospitals were selected from each city. Considering the feasibility of the study, the selected hospitals should have administrators who are members of the orthopedic Nursing Professional Committee of the Chinese Nursing Association. In the third stage, the investigators of each hospital were responsible for including patients and collecting data in orthopedic wards according to inclusion criteria and exclusion criteria.

### Participants

We defined older patients as aged ≥ 60 years. Inclusion criteria: (1) People aged ≥ 60 years; (2) Patients diagnosed with osteoporotic fracture and undergoing (hip/vertebral/humerus/radius) osteoporotic fracture surgery; (3) Patients in stable condition after surgery, in the recovery period, and not yet discharged from the hospital. (4) The first fracture and recurrent fracture were fractures caused by non-violent factors (e.g. fall, daily activities or minor trauma) and supported by clinical records (e.g. surgical reports, clinical records, previous or current X-rays). (5) The person agreed and cooperated to participate in the research and could understand the researcher's instructions. Exclusion criteria: (1) Pathological fracture (pathological fracture caused by malignant tumour, tuberculosis and other bone metabolic diseases that can cause fracture); (2) Serious traumatic fracture; (3) People with combined mental disorders; (4) Patients with combined other bone diseases such as osteoarthrosis; (5) People with severe anxiety, depression, and other emotions unable to cooperate in completing the questionnaire. This study was approved by the Ethics Committee of Chinese PLA General Hospital (No. S2023-621-01). The Orthopedic Nursing Professional Committee has designated personnel responsible for data collection, and has obtained ethical approval from various hospitals before data collection. All methods in this research were performed in accordance with the Declaration of Helsinki.

### Variables

Variables analyzed as risk factors for subsequent fracture were derived from clinical risk factors (CRFs) that may have an independent effect on fracture risk in clinical practice and literature studies. (1) General Information: age, BMI, comorbidity, trauma history, surgical history, family history of osteoporosis, smoking, alcohol consumption, occupation, education, marital status, average annual household income, and characteristics of the hospital where the patient was admitted (e.g. hospital level, hospital type, mode of admission). Trauma history mainly includes musculoskeletal injury caused by falls or other reasons. Surgical history refers to the history of orthopedic surgery in this study. (2) Characteristics of index fracture: fracture site, cause of fracture, BMD T-score. (3) Prevention information after index fracture: use of AOM, Use of calcium or vitamin D, fall risk assessment, fracture risk assessment, walking aids, fall prevention health education.

### Sample size

The sample size for Logistic regression was calculated empirically and we took 10 to 20 times of the risk factors. There were 35 suspected potential factors. Assuming a non-response rate of 10% for the study population, then the minimum sample size of events required was 385–770. The actual number of patients included was 9212.

### Statistical analysis

Data with > 30% missing information were excluded in our study. All variables that were to be included in the regression analysis were used in the imputation process. Then, duplicates and orphaned data were removed and irrational data were corrected.

In this study, a comprehensive statistical analysis approach was utilized to ensure the robustness and reliability of our predictive model. Initially, data analysis was conducted using SPSS 25.0 for quantitative and categorical data. Quantitative data were described using mean ± SD and analyzed with independent samples t-tests to compare differences across groups. Categorical data were expressed as percentages and subjected to χ2 tests, Fisher's exact test, or contingency tables analysis, as deemed appropriate.

Further analytical depth was added by including CRFs in univariate analyses to calculate ORs for various risk factors, identifying those significant with a P-value less than 0.05. These significant variables were subsequently incorporated into a multivariate logistic regression model to pinpoint independent risk factors, employing R 4.1.3. Internal validation of the model was further strengthened through 1000 bootstrap resamplings, aiming to estimate Harrell's C-index. This provided a quantitative measure of the model's predictive performance, reinforcing the validity of our findings.

Additionally, the discriminative ability of the model was evaluated by calculating the Area Under the ROC curve (AUC). The AUC assess the model's sensitivity and specificity at different thresholds, while the overall classification accuracy offered a comprehensive measure of the model's overall ability to correctly classify samples within the CV framework. In statistical terms, a P-value less than 0.05 was adopted as the criterion for significant differences, ensuring that improvements in model performance were statistically significant.

### Ethics approval statement

This study was approved by the Ethics Committee of Chinese PLA General Hospital (No. S2023-621-01). All methods in this research were performed in accordance with the Declaration of Helsinki.

### Consent for publication

The informed consents were obtained from participants.

## Data Availability

The data could be obtained from corresponding author.
